# A comparison across non-model animals suggests an optimal sequencing depth for *de novo* transcriptome assembly

**DOI:** 10.1186/1471-2164-14-167

**Published:** 2013-03-12

**Authors:** Warren R Francis, Lynne M Christianson, Rainer Kiko, Meghan L Powers, Nathan C Shaner, Steven H D Haddock

**Affiliations:** 1 Monterey Bay Aquarium Research Institute, , 7700 Sandholdt Rd, Moss Landing, CA 95039, USA; 2 Department of Ocean Sciences, University of California Santa Cruz, Santa Cruz, CA, USA; 3 Helmholtz Center for Ocean Research Kiel, GEOMAR, , Hohenbergstr. 2, Kiel, 24105, Germany; 4 The Scintillon Institute, , 9924 Mesa Rim Rd., San Diego, CA, 92121, USA

## Abstract

**Background:**

The lack of genomic resources can present challenges for studies of non-model organisms. Transcriptome sequencing offers an attractive method to gather information about genes and gene expression without the need for a reference genome. However, it is unclear what sequencing depth is adequate to assemble the transcriptome de novo for these purposes.

**Results:**

We assembled transcriptomes of animals from six different phyla (Annelids, Arthropods, Chordates, Cnidarians, Ctenophores, and Molluscs) at regular increments of reads using Velvet/Oases and Trinity to determine how read count affects the assembly. This included an assembly of mouse heart reads because we could compare those against the reference genome that is available. We found qualitative differences in the assemblies of whole-animals versus tissues. With increasing reads, whole-animal assemblies show rapid increase of transcripts and discovery of conserved genes, while single-tissue assemblies show a slower discovery of conserved genes though the assembled transcripts were often longer. A deeper examination of the mouse assemblies shows that with more reads, assembly errors become more frequent but such errors can be mitigated with more stringent assembly parameters.

**Conclusions:**

These assembly trends suggest that representative assemblies are generated with as few as 20 million reads for tissue samples and 30 million reads for whole-animals for RNA-level coverage. These depths provide a good balance between coverage and noise. Beyond 60 million reads, the discovery of new genes is low and sequencing errors of highly-expressed genes are likely to accumulate. Finally, siphonophores (polymorphic Cnidarians) are an exception and possibly require alternate assembly strategies.

## Background

RNA-seq has provided a powerful tool for analysis of transcriptomes. For non-model organisms with limited genomic information, transcriptome sequencing provides a cost-saving tool by only sequencing functional and protein coding RNAs, thus providing direct information about the genes [1]. There are many benefits of sequencing a genome, but for relatively large genomes such as human and mouse, protein coding regions account for under 5%, thus most of the sequencing effort would go to sequencing either regulatory regions or repetitive elements [2]. Smaller genomes could be sequenced and assembled to complement the transcriptomes, though this is not a tractable approach if a genome is quite large. Even still, de novo genome assembly can produce errors by itself [3].

Despite its advantage, transcriptome assembly does present additional challenges when compared to genome assembly. Unlike genomes where most sequences should be approximately equally represented, coverage of any given sequence in a transcriptome can vary over several orders of magnitude due to expression differences [4]. Because coverage can vary, there is also a question of sequencing depth. Theoretically, there is a sequencing depth beyond which addition of more reads does not provide new information, known as the saturation depth. Several studies have used approaches which map reads onto reference genomes and these have suggested saturation depths at 95% gene coverage ranging from 1.2 million reads to 50 million for mRNA level coverage, and up to 700 million for splice variants [5-7]. However, these studies all made use of short reads around 36bp and were not assembling the transcriptomes de novo.

Several recent studies have already made use of next-generation sequencing reads for de novo transcriptome assembly [8-15]. The number of reads used for assembly in these studies varies widely, ranging from 2.6 million reads up to 106 million reads [10,11]. The assembly strategies are equally varied, but share the initial step of removing low-quality reads and adapters whereupon all remaining reads are assembled. The assembly quality estimates vary as well with the most common measure of quality based on BLAST hits to public databases like Uniprot, though it was noted that under-representation of many taxa in public databases limits this approach [8].

While many parameters must be optimized for the specific assembly, it is both inconvenient and costly to acquire more reads by resequencing. Presently, there is no clear consensus of what sequencing depth is optimal or what factors would contribute to the adequate depth. The problems of omitted genes or variants are obvious with too few reads. On the other hand, it was suggested that greater depth may create errors in differential expression analyses, cost more, and take longer to assemble [16]. Thus, here we use the same assembly strategy across a diverse set of organisms to isolate the effects of read count on assembly quality to attain a general estimate of optimal read count. We compare trends from de novo assemblies across six phyla. These animals include the mouse (used as a control for the non-model samples), the Humboldt squid *Dosidicus gigas*, the scaleworm *Harmothoe imbricata*, the decapod *Sergestes similis*, the copepod *Pleuromamma robusta*, the ctenophore *Hormiphora californensis*, and the siphonophore *Chuniphyes multidentata*. To our knowledge, this is the first study to suggest an optimal number of reads for de novo assembly for the purposes of mRNA level analysis. These results are applicable to studies of organisms with limited genomic resources.

## Results and discussion

### De novo assembly of transcriptomes

#### Assembly of mouse heart transcriptome

Raw mouse-transcriptome reads from the ENCODE project were downloaded from NCBI short-read archive. Sample SRR453174 (mouse heart RNA-seq) consisted of 82,886,668 x76bp reads as paired-ends. Filtration (see Methods) removed 11.7% of the reads, almost 95% of which were due to low quality scores. In order to examine the role of number of reads on the assembly, we computationally sub-sampled randomized sets from the original library. It is suggested that sequencing of very small numbers of reads can be most subject to biases and that cDNA normalization can improve the uniformity of the library at low numbers of reads [17]. Such an approach might be quite costly, and the computational sub-sampling approach has the advantage of drawing from the largest pool of reads and avoid biases which could occur at low numbers of reads. Subsets of the filtered library were generated containing 1,5,10,20,30,40,50,60, and 70 million reads. Reads from each set were included in the next largest set, thus all of the reads in the 1 million set are included in the 5 million read set, and so forth. These sets were assembled with Velvet/Oases [18,19] and Trinity [20] (For a detailed comparison of assemblers, see [21]).

Schulz et al. reported reliable parameters for Oases which produced high-quality assemblies of mouse and human cell cultures, using 64 million and 30 million reads, respectively [19]. This included use of a broad k-mer range with a low starting k-mer of 19 or 21 up to a k-mer of 33 or 35. Accordingly we used k-mers from 21 to 33. Also, a minimum k-mer coverage is required by Oases to retain any given node during the assembly process; by default this is 3 in Oases, that is, any node must have at least three-fold coverage for that node to be used. Some differences were observed in the output when this parameter was changed, and so the same data were assembled with coverage cutoff of 3 (referred to hereafter as C3) and a stricter cutoff of 10 (C10).

**Figure 1:**
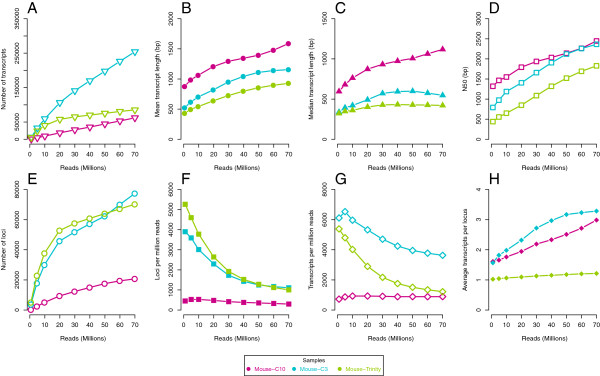
**Assembly metrics for mouse heart transcriptome. **Assorted size metrics for the mouse heart transcriptome showing (**A**) number of transcripts; (**B**) mean length; (**C**) median length; (**D**) N50 of the assembly; (**E**) number of loci; (**F**) loci per million reads; (**G**) transcripts per million reads; (**H**) transcripts per locus.

The number of transcripts (Oases terminology for contigs) increases steadily for all assemblies (Figure 1A). C10 also had substantially fewer transcripts and accordingly much higher mean and median lengths (Figure 1B-D). The pattern of increase for median and N50 (length for which half of the total bases are in contigs of this length or longer) tracked the mean for the C10 assembly, but not the C3 assembly which did not have a clear qualitative pattern. The mean, median and N50 were all lower for the Trinity assembly than the C3 despite having far fewer contigs.

Oases generates transcript “loci”, which is Oases terminology for the de-Brujin graph clusters meant to represent genes and their splice variants or highly-similar paralogs. Both curves approach to a plateau for locus counts (Figure 1E-F). The greatest increase in loci was between using 10 million to 20 million reads for both C3 and C10. Similarly, the C3 assembly shows a decrease in the number of transcripts per read (Figure 1G), while the C10 assembly shows an almost constant number of transcripts per read. The number of transcripts increases while the number of loci tend to level off and this means the number of transcripts per locus always increases with more reads (Figure 1H). That is, on average, more variants will be generated with more reads even though some of these are likely due to noise. While the Trinity assembly more closely matches the trends for transcripts per read of the C3, the “components” (closest obvious parallel of loci) remain close to a unit ratio, suggesting that most components have only one associated sequence.

#### Assembly of invertebrate transcriptomes

**Figure 2:**
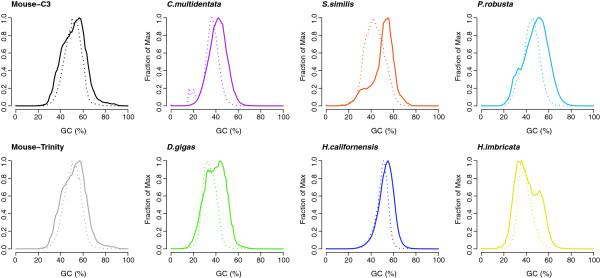
**Histograms of GC distributions. **Dashed lines show the normalized abundance of transcripts by GC content, while solid lines show normalized abundance of the raw reads.

Transcriptomes across a broad range of taxa were assembled as with the mouse and statistics of the largest assemblies are presented in Table 1. The stated GC content of the mouse genome is 42% while a subset of conserved genes showed a much higher value of 51.24% [22,23]. Interestingly, for all assemblies except for mouse, the average GC content of the assembled contigs was lower than that of the raw reads (Figure 2), suggesting either that certain genes contribute much more to the overall GC content of the library or that biases can be introduced from the assembly.

**Table 1:** Assembly Statistics

**Organism**	**Mouse cov-cutoff-3**	**Mouse cov-cutoff-10**	**Mouse-Trinity**	**Chuniphyesmultidentata**	**Sergestessimilis**	**Pleuromamma robusta**	**Dosidicusgigas**	**Hormiphoracalifornensis**	**Harmothoeimbricata**
Phylum	Chordata	Chordata	Chordata	Cnidaria	Arthropoda	Arthropoda	Mollusca	Ctenophora	Annelida
Tissue	Heart	Heart	Heart	Whole body	Legs	Whole body	Mantle	Whole body	Scale
Raw Reads	82,886,668	82,886,668	82,886,668	103,415,276	93,597,558	64,116,306	60,661,588	64,675,964	75,608,018
Raw GC (%)	51.90	51.90	51.90	42.29	50.74	48.86	39.89	53.71	41.52
Filtered Reads	73,187,048	73,187,048	73,187,048	102,366,438	92,423,904	63,867,922	56,264,099	57,583,204	70,340,105
Assembled Reads	70,000,000	70,000,000	70,000,000	80,000,000	80,000,000	63,867,922	56,264,099	57,583,204	70,340,105
Transcripts	254,215	62,353	85,294	338,254	107,082	196,104	86,897	175,701	191,290
Total Length (Mbp)	293.55	98.84	79.12	314.99	159.59	240.05	143.09	272.23	216.66
Mean (bp)	1,154	1,585	927	931	1,490	1,224	1,646	1,549	1,132
Median (bp)	547	1,119	421	421	837	855	1,026	1,153	689
N50 (bp)	2,364	2,447	1,828	1,854	2,803	1,993	2,876	2,373	1,949
Oases Loci	77,411	20,889	70272	49,831	18,139	22,385	14,227	17,960	21,914
GC (%)	54.08	53.95	53.46	31.24	44.66	45.78	36.55	51.66	40.53

Summary statistics of the largest transcriptome assembly for each organism.

**Figure 3:**
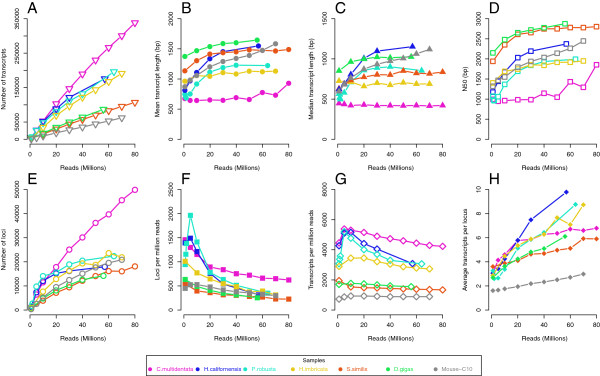
**Assembly metrics for marine organisms. **Assorted size metrics as in Figure 1; (**A**) number of transcripts; (**B**) mean length; (**C**) median length; (**D**) N50 of the assembly; (**E**) number of loci; (**F**) loci per million reads; (**G**) transcripts per million reads; (**H**) transcripts per locus. Purple - *C. multidentata*; blue - *H. californensis*; teal - *P. robusta*; green - *D. gigas*; yellow - *H. imbricata*; red - *S. similis*. For comparison, C10 mouse data are shown in gray.

For three of six samples (*D.gigas*, *H.imbricata *and *S.similis*), only select tissues were used for RNA extraction while the rest were whole body (*C.multidentata*, *H.californensis *and *P.robusta*. It should be noted that the *C.multidentata *sample combined sequences from the two major tissues, siphosome and nectophore and that the *P.robusta *sample was a combination of multiple individuals. This decision was based on size of the animals since very small organisms are difficult to dissect. Assembly trends analogous to Figure 1 for the six animals are shown in Figure 3. Mouse C10 data from Figure 1 are shown in gray as reference. Three main trends emerged. Whole-body samples were characterized by a rapid gain of transcripts and increases in transcript size through 40 million reads, while all other parameters level off after 40 million reads. Single tissue samples showed a slow gain of relatively long transcripts across fewer loci. Lastly, the whole-body siphonophore showed continuous gain of both short transcripts and loci without reaching an asymptote at the maximum number of reads assembled.

Four of the animals showed modest gains in mean, median and N50 with more reads (average 20% from fewest to most reads), while *P.robusta *and *H.californensis* nearly doubled from the fewest to the most reads (Figure 3B-D). Most of the transcript-length increase occurred before 30 million reads, suggesting that adding more reads did not produce longer sequences beyond that threshold, or that they became longer at the same rate that new, short transcripts were generated. As with the mouse samples, transcripts were added continually with more reads (Figure 3A). Compared to the mouse, on average these six animals all had more transcripts per locus (Figure 3H). It is unclear why this would be the case, though the C10 assembly had the fewest number of transcripts overall for all numbers of reads. The most pronounced gains in loci happened within the first 10 million reads, particularly for *P.robusta *and *H.californensis *(Figure 3E-F). Gains in loci tended to level out between 40 and 60 million reads, suggesting most genes (or parts of genes) were assembled by 60 million reads.

A very high number of transcripts for *C.multidentata *(Figure 3, purple) led to the lowest mean, median, and N50. The number of removed, low-quality reads is comparable in this sample to others, so low quality is unlikely to be the cause. As two sets of reads were combined into a whole animal, this may have created artifacts. However, another *C.multidentata *siphosome sample produced assemblies with large numbers of relatively short sequences (data unpublished). One possible explanation is that siphonophores have continuously developing differentiated zooids [24]. These zooids have specialized functions which are in some ways analogous to organs, and a whole organism can contain multiple developmental stages and express a large part of the genome, possibly confounding the assembly process. Assemblies of a number other siphonophores (data unpublished) similarly had many short transcripts. We speculate that alternate assembly strategies or very careful dissections might be required for animals in this lineage.

### Discovery of conserved genes

#### Conserved mouse genes

**Figure 4:**
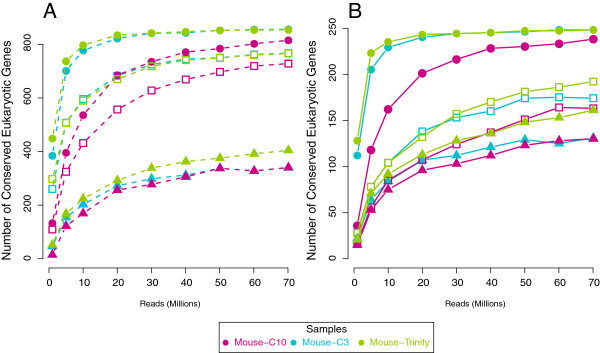
**Conserved genes in the mouse transcriptome. **Saturation curves of discovery of genes in the mouse heart from a set of (**A**) 860 conserved orthologs from NCBI and (**B**) a subset of 248 conserved orthologs; genes which have any blast hit are shown in circles; genes which the translated protein was within the expected size range of the conserved gene are in squares; proteins which are 100% identical to a canonical protein in Uniprot/Swissprot mouse database are shown in triangles.

One approach used to assess genome completeness is to search only for conserved eukaryotic orthologous genes (KOGs). The current NCBI KOG database has 860 gene clusters across 7 eukaryotes with over 16000 proteins [25]. The KOG reference genes did not include mouse sequences, and this provided an opportunity to test predictions about de novo transcriptome quality while still having a reference in the end to confirm the reliability of the sequences. For each KOG, the transcripts were aligned against the reference KOGs with tblastn, and the best coding sequence was kept. The putative proteins were classified by length relative to the range of sizes of the reference KOGs. The size range allowed some flexibility, as 12 mouse proteins were larger than the longest reference protein for that KOG, and 5 were shorter than the shortest reference protein. Finally the proteins were aligned with blastp against reviewed mouse proteins in Uniprot to determine accuracy. One protein was unreviewed (Q3UWL8, Mouse Prefoldin 4). For this test, Trinity and Oases are comparable at assembling full-length proteins, though Trinity appears to be slightly better at reconstructing canonical proteins (Figure 4A).

However, gene duplications present difficulties for such assessments unless one had a priori knowledge of how many copies should be present in the genome. For this study, we also used the subset of eukaryotic KOGs containing 248 genes from the CEGMA pipeline which were identified as single-copy orthologs in most genomes [26,27]. Almost one third of these KOGs are involved in processes like transcription and translation and were expected to be expressed in many tissues. Trinity and Oases with a lower coverage cutoff of 3 found similar numbers of KOGs at much lower numbers of reads (Figure 4B) than compared to the C10 assembly. Also more KOGs were found within expected length much faster with C3 than with the higher cutoff of 10, and the Trinity assembly outperformed both of these. These results suggest that it is better to have a lower cutoff and assemble more sequences. Likewise, the Trinity assembly had more transcripts than C10 and were shorter than those in C3, yet more KOGs were found with fewer reads and more coding transcripts were correctly assembled at greater numbers of reads. However, for the Oases assemblies this had remarkably little effect on the number of correct canonical proteins that were found (Figure 4, triangles). Although there is some overestimation, no protein designated as too short was ever correct. Regarding the fate of the other full-length proteins, for C3 at 70 million reads, 186 KOGs were found within the expected range, though only 131 were correct. Eight of the 186 KOGs had only 1 mismatch in the amino-acid sequence compared to the reference protein which could be due to errors, splice variants, tissue-specific modifications or alleles. The remaining KOGs had at least two amino-acid changes but were within the size range. Thus for the mouse, the size range was a reliable predictor of true full-length proteins.

#### Conserved invertebrate genes

**Figure 5:**
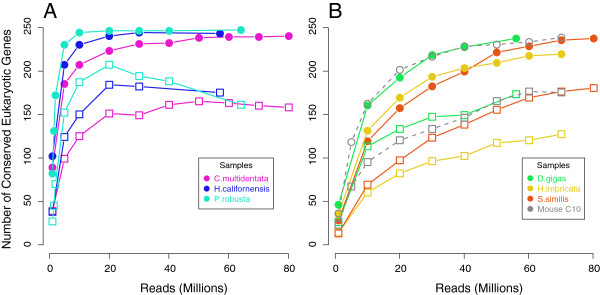
**Conserved genes in marine organisms. **As in Figure 4, genes with a reliable blast hit are shown in circles for all 6 marine organisms; genes which the translated protein was within the expected size range of the conserved gene are in squares. Purple - *C. multidentata*; blue - *H. californensis*; teal - *P. robusta*; green - *D. gigas*; yellow - *H. imbricata*; red - *S. similis*. For comparison, C10 mouse data are shown in gray.

We then examined our invertebrate transcriptomes for completion using the same set of KOGs. There was a clear, qualitative difference between whole-body organisms (Figure 5A) and dissected tissues (Figure 5B). C10 mouse data are included for reference. For whole-body transcriptomes, over 90% of the KOGs were detectable at 20 million reads, yet the number of within-length KOGs went down with higher numbers of reads past 20 million. This could be caused if proteins declared to be within-range were longer than the true protein due to mis-assembly causing addition of pieces, or if the true protein became mis-assembled with addition of noisy reads. In nearly all of our assemblies, it was the latter: mis-assembly of the putative protein which generated stop codons. *C.multidentata *(Figure 5A, purple) was again exceptional, as the number of within-length KOGs increased more slowly with addition of more reads than the other two whole-body animals (*H.californensis *and *P.robusta*) and only decreased after 50 million reads rather than 20 million.

For dissected-tissue transcriptomes (*Dosidicus gigas*, *Harmothoe imbricata*, and *Sergestes similis*), the rate of discovery of KOGs was much slower with between 63% and 81% of KOGs detectable at 20 million reads (Figure 5B). This was not surprising since those genes may not be highly-expressed in all tissues and it is likely tissue-specific genes account for the bulk of the assembly at low numbers of reads. Isolated tissues may express fewer universal KOGs that we selected in our test, and we expected that other abundant transcripts should mis-assemble at high numbers of reads in that tissue. However, the dissected-tissue transcriptomes had longer transcripts and fewer loci, suggesting this was not the case. Since whole-animal transcriptomes include all tissues, a greater proportion of the genome is expressed so coverage of any given transcript or splice-variant is proportionally much lower. The length saturation patterns appear to be different between whole-animal and tissue transcriptomes. However, using conserved genes as a metric, there appears to be limited benefit of sequencing beyond 60 million reads.

### Mis-assembly at high numbers of reads

KOGs with single-exon coding sequences in the mouse were examined for mis-assembly. To increase the number of genes examined, another set of KOGs from only metazoans (*C.elegans*, *D.melanogaster *and *H.sapiens*, CDH) was used. The KOG database at NCBI contained 1147 clusters common to CDH. Again, only genes that were annotated as single copy in all three animals were used, leaving a final set of 202 KOGs specific to metazoans. These combined sets of 450 had 12 genes in mouse which were presumed single-copy and annotated in NCBI to have a single-exon coding sequence (GenBank:NP_062724.1, NP_666327.2, NP_082281.2, NP_058612.3, XP_899832.1, NP_001153802.1, NP_001104758.1, NP_077152.1, XP_486217.2, NP_598737.1, NP_032025.2, NP_075969.1). At 70 million reads, 3 genes in C3 had alternate erroneous coding sequences: NAT6, CHMP1B1/DID2, FTSJ (N-acetyl transferase 6, Charged multivesicular body protein 1b-1, Ribosomal RNA methyltransferase, respectively). The sequence of CHMP1B1 was never assembled correctly for any number of reads and the best version was missing 9 amino acids at the N-terminus including the start codon. Only NAT6 had extraneous coding sequence in C10, suggesting that such errors can be controlled by limiting read count as well as increasing k-mer coverage thresholds.

**Figure 6:**
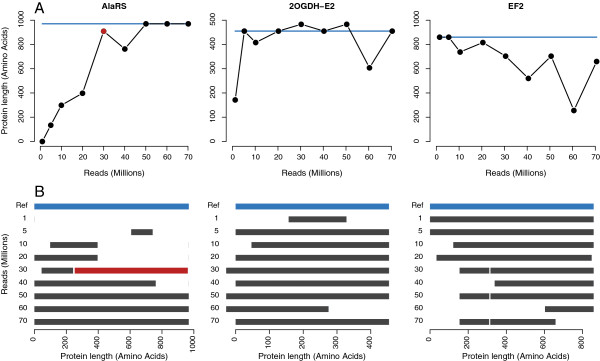
**Selected cases of misassembly. **Orthologs were tracked across multiple sequencing depths, and selected examples are here showing some of the pitfalls of assembly. (**A**) The lengths of three proteins are shown (AlaRS, Alanyl-tRNA synthetase; 2-OGDH-E2, 2-oxoglutarate dehydrogenase subunit E2; EF2, Elongation factor 2), and the canonical protein length is indicated by a blue line. (**B**) Protein alignment view of the same three proteins compared to the Uniprot/Swissprot canonical protein, which is shown as the blue bar. A chimeric portion of AlaRS at 30 million reads is indicated by the red bar, where it contains a sequence from the putative mitochondrial alanyl-tRNA synthetase 2 protein (NP_941010), and corresponds to the red point at 30 in (A). For AlaRS and EF2, some alignments produced a few short gaps compared to the reference proteins.

While some mis-assemblies can occur with more reads, overall this is not a problem, as shown by the curves in Figures 4 and 5. However, select cases of mis-assembly of the mouse genes are shown in Figure 6. AlaRS (Alanyl-tRNA synthetase) presents an example of the optimal scenario, whereby the protein is not found at all with few reads, but then pieces come together with the addition of more reads until the final protein is correctly assembled. The majority of proteins follow this trend. 2-OGDH shows an unusual oscillation between the reference protein and alternate forms. EF2 is assembled correctly with few reads, then errors accumulate as more reads are added. From this, it cannot be assumed that the largest set of reads will produce the best contigs. Schulz et al. indicated that between 10 and 20% of Oases transcripts had some degree of misassembly [19]. This value was found to correlate with the smallest k-mer used in assembly and the authors suggest using larger k-mers if problems arise due to chimeric transcripts. Thus if using more reads, it may be advisable to use larger k-mers or a higher static coverage cutoff.

## Conclusions

In this study, number of whole animals and tissues from non-model organisms and one mouse organ were assembled and the completeness was assessed using a set of conserved genes. Additionally, a comparison was made between two high-performing assemblers with respect to the mouse data. Oases required much greater memory usage while Trinity had much longer run times (approximately 2-fold longer). Both Trinity and Oases perform comparably at assembling conserved genes across a large set, indicating that the saturation depth is not greatly affected by assembler choice.

Overall, these results suggest that for whole-body transcriptomes and individual organs or cells, 30 and 20 million reads are sufficient for mRNA level coverage, respectively. For the read length used in this study, that would produce 2-3 gigabases of sequence. It should be noted that the mouse data consisted of shorter reads than used for the invertebrates, but this did not appear to have substantial effect as this difference was only between 75bp reads and 100bp reads. Assembly errors are evident in whole-body transcriptomes after 30 million reads, and the average length appeared to level off at the same depth. Presumably this depth would apply for studies of differential expression as well, as the highly expressed transcripts should be present and distinguishable at that sequencing depth. In our experience, we find it is optimal to acquire between 50 and 60 million reads, and then sub-sample up around 20 or 30 million. This approach reliably assembles nearly all proteins of interest. There are still observable differences between assemblies, although some of these differences may ultimately be due to variations in RNA quality or properties of the animal.

## Methods

### Samples and sequencing

*D.gigas *and *H.californensis *were collected in the Gulf of California by jig and trawl net, respectively. *C.multidentata *and *S.similis *were collected in the Monterey Bay using remotely-operated-underwater vehicles. *H.imbricata* samples were given courtesy of T. Rivers. All samples were flash frozen in liquid nitrogen immediately following collection. Total RNA was extracted using RNeasy kit (Qiagen) as per instructions. *C.multidentata *RNA was extracted with Trizol and purified with the RNeasy kit. Preparation of RNA-seq libraries was done using Illumina TruSeq kit for paired end reads. Total RNA was sent for sequencing at University of Utah. Multiple individuals of *P.robusta *were sampled off the coast of Namibia and sequenced at the Institute for Clinical Molecular Biology, (IKMB, Kiel University). Sequencing was done using the Illumina HiSeq2000 platform on a paired-end protocol with 100 cycles. Mouse heart data were downloaded from NCBI accession GSE36025, sample SRR453174.

### Transcriptome assembly

All computations were done on a computer with two quad-core processors and 96GB RAM. For each sample, the orders of all raw reads were randomized with the randomize.cpp program and processed with a modified version of the filter_illumina.cpp program in the Agalma transcriptome package (https://github.com/caseywdunn/agalma). This removed low-quality reads (with mean Phred score < 28), as well as reads containing adapters and reads that were mostly repeated bases, such as polyT tracts. Reads from pairs with one good read and one bad read retained the good read for the largest assembly. Otherwise, only good pairs were used in other assemblies. The transcriptome for each set was assembled de novo using Velvet v1.2.06 /Oases v0.2.06. Identical assembly parameters were used unless otherwise noted. Multiple k-mer assemblies were generated (21,25,29,33) and merged with Oases-M (k-mer of 27). A static coverage cutoff of 10 was used and insert size of the paired ends was estimated with the “-exp_cov auto” parameter, typically around 180bp, as expected. The minimum contig length was set to 100, which is the read length. The Trinity assembler was also used for comparison of mouse assemblies using the same filtered subsets of reads. Other than insert length being specified as the upper limit rather than the mean, default assembly parameters were used including a minimum transcript length of 200bp. Transcript lengths and GC content were measured with an in-house python script, sizecutter.py, available at the MBARI public repository (http://bitbucket.org/beroe/mbari-public/src).

### Conserved gene analyses

All blast searches were done using the NCBI blast 2.2.25+ package [28]. We generated a script to blast and analyze the matches, kogblaster.py (on the public repository, as above). Briefly, the reference KOGs (860 orthologous groups from NCBI, or 248 orthologous groups, from http://korflab.ucdavis.edu/Datasets/cegma/) were aligned to each assembly with tblastn with an e-value cutoff of 10^-6^. For each alignment, the subject hit was translated and coding sequences were only kept if they contained both start and stop codons. From this subset, the best alignment was declared to be the correct sequence. Next, the length of the correct sequence was used to estimate whether that sequence was full-length relative to the conserved orthologs. For each KOG in the CEGMA dataset, there were 6 proteins from 6 species and there was some variability in protein length (average 11.8% from longest to shortest). The variability from the the reference set was used to establish boundaries for size classifications which were made to watch the progression of assembly of individual genes: (1) within the size range of the KOG; (2) within the range but where the alignment was less than 90% of the length of the protein; (3) longer than those in the size range; (4) shorter than the size range; (5) shorter than the size range and shorter than the alignment, often indicative of a stop codon bridged by the alignment. The full-length size range was defined by ratios of the shortest protein to the second shortest, and analogously for the longest protein and second longest. For example, if the shortest protein within a KOG was 80AAs, and the second shortest was 100AAs, the lower bound would be (80∗(80/100)), and thus 64AAs. This was calculated for each KOG, and was to account for proteins which could potentially become the ‘new’ shortest or longest. Ultimately, only those within the size range (1) were declared as full-length sequences.

The animals in this study were treated ethically and responsibly. Because no vertebrates or octopus were involved, no formal certification is required per the Helsinki Declaration. The mouse data presented in the paper were not obtained from our experiments, but were downloaded from a database.

## Competing interests

The authors declare no competing financial interests.

## Authors’ contributions

WF, RK and SH designed experiments. LC, RK, MP and SH caught animals. LC, RK, MP and NS processed animals and extracted RNA. WF assembled transcriptomes. WF, RK and SH analyzed data. WF wrote the paper. All authors read and approved the final manuscript.
